# Optimization of the expression, purification and polymerase activity reaction conditions of recombinant human PrimPol

**DOI:** 10.1371/journal.pone.0184489

**Published:** 2017-09-13

**Authors:** Elizaveta O. Boldinova, Gorazd Stojkovič, Rafil Khairullin, Sjoerd Wanrooij, Alena V. Makarova

**Affiliations:** 1 Institute of Molecular Genetics of Russian Academy of Sciences, Kurchatov sq. 2, Moscow, Russia; 2 Moscow State University of Fine Chemical Technologies, Vernadsky Prospect 78, Moscow, Russia; 3 Department of Medical Biochemistry and Biophysics, Umeå University, Umeå, Sweden; 4 Institute of Fundamental Medicine and Biology, Kazan (Volga Region) Federal University, K.Marx, 18 Kazan, Russia; Istituto di Genetica Molecolare, ITALY

## Abstract

Human PrimPol is a DNA primase/polymerase involved in DNA damage tolerance and prevents nuclear genome instability. PrimPol is also localized to the mitochondria, but its precise function in mitochondrial DNA maintenance has remained elusive. PrimPol works both as a translesion (TLS) polymerase and as the primase that restarts DNA replication after a lesion. However, the observed biochemical activities of PrimPol vary considerably between studies as a result of different reaction conditions used. To reveal the effects of reaction composition on PrimPol DNA polymerase activity, we tested the polymerase activity in the presence of various buffer agents, salt concentrations, pH values and metal cofactors. Additionally, the enzyme stability was analyzed under various conditions. We demonstrate that the reaction buffer with pH 6–6.5, low salt concentrations and 3 mM Mg^2+^ or 0.3–3 mM Mn^2+^ cofactor ions supports the highest DNA polymerase activity of human PrimPol *in vitro*. The DNA polymerase activity of PrimPol was found to be stable after multiple freeze-thaw cycles and prolonged protein incubation on ice. However, rapid heat-inactivation of the enzyme was observed at 37ºC. We also for the first time describe the purification of human PrimPol from a human cell line and compare the benefits of this approach to the expression in *Escherichia coli* and in *Saccharomyces cerevisiae* cells. Our results show that active PrimPol can be purified from *E*. *coli* and human suspension cell line in high quantities and that the activity of the purified enzyme is similar in both expression systems. Conversely, the yield of full-length protein expressed in *S*. *cerevisiae* was considerably lower and this system is therefore not recommended for expression of full-length recombinant human PrimPol.

## Introduction

PrimPol is a unique DNA polymerase with primase activity [1–3]. Human PrimPol is a 560 a.a. protein encoded by the *CCDC111 (PRIMPOL)* gene and belongs to the archaeo-eukaryotic primase (AEP) superfamily [4]. PrimPol homologs were found in many eukaryotes including animals, plants, fungi and protists [2, 5]. *In vitro*, human PrimPol works as a translesion DNA polymerase and can bypass DNA lesions such as 8-oxo-G and pyrimidine-photoproducts [1, 2, 6, 7]. Similarly to other translesion DNA polymerases, PrimPol lacks the 3'-5'-exonuclease activity and exhibits low fidelity of DNA synthesis [6, 8]. In addition, PrimPol is only the second primase found in human cells along with the Pol α-primase complex. However, unlike Pol α-primase PrimPol can synthesize primers using deoxyribonucleotides during *de novo* DNA synthesis [1, 2]. PrimPol can utilize both Mg^2+^ and Mn^2+^ ions as cofactors for both priming and polymerase activities [2, 6].

PrimPol plays an important role in the maintenance of genome stability by protecting the cells from replication stress induced by DNA damage, and by assisting fork progression on undamaged DNA. During the nuclear genome replication, PrimPol promotes stalled replication fork progression by re-initiating the DNA synthesis downstream of DNA damage and non-B DNA structures [7, 9]. Importantly, PrimPol was also found in mitochondria, making it only the second DNA polymerase found in mitochondria after Pol γ [2]. PrimPol functionally interacts with the mitochondrial replicative helicase TWNK (Twinkle) [10] and human cells deficient in PrimPol display impaired mitochondrial replication [2]. Nonetheless, the exact role of this polymerase in mitochondria remains to be established, since the addition of human PrimPol to the *in vitro* reconstituted mitochondrial replication fork does not promote the synthesis past oxidative DNA damage [10].

Several groups reported biochemical studies of PrimPol and often this polymerase demonstrated low catalytic activity, requiring significant excess of protein over DNA template in the assays. It was shown that PrimPol has relatively low binding affinity to primer/template DNA substrate and to incoming dNTPs [6, 8, 11]. Whereas poor catalytic activity might be an intrinsic characteristic of PrimPol, the composition of the reaction buffer, protein folding in the expression system and protein instability may also contribute to the low catalytic activities observed.

In this work, we have tested the PrimPol DNA polymerase activity under different reaction conditions and measured its stability during incubation at different temperatures as well as after freeze-thaw cycles and mechanical shear stress. Additionally, we tested its expression in five different expression systems in order to reveal the optimal method of recombinant human PrimPol production. As a result, we describe the optimal conditions for *in vitro* analysis of PrimPol activity.

## Materials and methods

### Strains and cell cultures

Three *E*. *coli* and one *S*. *cerevisiae* strains were used in this study (Table 1). *E*. *coli* strains carry a DE3 lysogen that enables expression of T7 RNA polymerase. FreeStyle™ 293-F cells (Invitrogen) were used for transient expression of PrimPol in human suspension cell culture.

**Table 1:** 

Host organism	Strain	Genotype	Key feature	Source
*E*. *coli*	BL21(DE3)	F^−^*ompT hsdSB*(r_B_−m_B_−) *gal dcm (DE3)*	- deficient in Lon and OmpT proteases	Novagen
	Rosetta 2 (DE3)-pRARE2	F^−^*ompT hsdSB*(r_B_−m_B_−) *gal dcm (DE3)* [pRARE2 Cam^*R*^]	- deficient in Lon and OmpT proteases - encodes tRNAs for seven codons rarely used in *E*. *coli* (AUA, AGG, AGA, CUA, CCC, GGA and CGG) to enhance the expression of eukaryotic proteins	Novagen
	ArcticExpress(DE3)-pRIL	F^−^*ompT hsdS*(r_B_−m_B_−) *dcm*^+^ Tet^r^ *gal λ*(DE3) *endA* Hte [*cpn10 cpn60* Gent^r^] [*argU ileY leuW* Str^r^]	- deficient in Lon and OmpT proteases - encodes 3 tRNAs genes (*arg*, *ile* and *leu*) for codons rarely used in *E. coli* to enhance the expression of eukaryotic proteins - co-expresses the cold-adapted chaperons Cpn10 and Cpn60	Agilent Technologies
*S*. *cerevisiae*	BJ2168	*MATα*, *ura3-52*, *trp1-289*, *leu2-3*, *112*, *prb1-* *1122*, *prc1-407*, *pep4-3*	- deficient in proteases A, B and C	ATCC 208277
*human cells*	FreeStyle™ 293-F cells	HEK 293-derived cell line	- adapted to suspension culture in serum free media	Invitrogen

### Purification of PrimPol from *E*. *coli*

The full-length cDNA of the *CCDC111* human gene was obtained from Origene (http://www.origene.com). The PrimPol cDNA was PCR-amplified and cloned into the pGEX6P1 plasmid under the control of the *tac* promoter, in frame with an N-terminal GST-tag (26 kDa glutathione-S-transferase from *Schistosoma japonicum*) using the BamHI and SalI restriction sites. Three strains of *E*. *coli* cells were transformed with PrimPol-pGEXP1 and several colonies from fresh plates were used to make overnight start cultures at 37ºC with appropriate antibiotics. The start cultures were used to inoculate 2 liters of LB-medium at 1:100 dilution. The culture was grown at 22°C till 0D_600_ 0.4–0.5. At this point, the temperature was reduced till 19ºC for BL21(DE3) and Rosetta 2(DE3)-pRARE2 strains, and to 11–12ºC for ArcticExpress(DE3)-pRIL strain. Protein expression was induced by 0.5 mM IPTG and the cells were then cultivated for 19 h. Cells were harvested by centrifugation at 3500 x g for 30 min and frozen at -80ºC.

For PrimPol purification, the frozen cell pellets were thawed on ice and resuspended in 80 ml of 1x lysis buffer A (50 mM HEPES pH 7.4, 8% glycerol, 600 mM KCl, 20 mM K_2_HPO_4_/KH_2_PO_4_ pH 7.4, 1 mM DTT, 0.1% Tween 20, 10 µM pepstatin, 10 µM leupeptin, 2.5 mM benzamidine, 0.5 mM PMSF) and sonicated for 15 min. The lysates were centrifuged at 5000 g for 30 min and the cleared supernatants were gently agitated with 1 ml of glutathione-sepharose resin (GE Healthcare) for 4 h. The beads were packed into a disposable BioRad column and sequentially washed with 100 ml of lysis buffer A, 100 ml of wash buffer 1 (50 mM HEPES pH 7.6, 8% glycerol, 300 mM KCl, 15 mM K_2_HPO_4_/KH_2_PO_4_ pH 7.6, 1 mM DTT, 0.1% Tween 20, 0.5 mM PMSF) and 100 ml of wash buffer 2 (50 mM HEPES pH 7.8, 8% glycerol, 100 mM KCl, 10 mM K_2_HPO_4_/KH_2_PO_4_ pH 7.8, 1 mM DTT, 0.1% Tween 20, 0.5 mM PMSF). Proteins were eluted by three stepwise washes with 1 ml of elution buffer 1 (50 mM HEPES pH 8.0, 8% glycerol, 100 mM KCl, 10 mM K_2_HPO_4_/KH_2_PO_4_ pH 8.0, 1 mM DTT, 0.1% Tween 20, 30 mM of reduced glutathione).

As GST-fused PrimPol retained biochemical activity [this study and 2] the DNA polymerase activity of the protein produced in different *E*. *coli* strains was compared without removing the tag. In all other experiments, PrimPol produced in BL21(DE3) and ArcticExpress(DE3)-pRIL strains without the GST-tag was used unless indicated. To cleave the GST-tag, the fusion protein was incubated overnight at 4ºC with HRV 3C protease. To remove the free GST tag and the 3C protease after the cleavage, the proteins were diluted 1.3-fold with dilution buffer (30 mM Hepes pH 7.4, 8% glycerol, 1 mM DTT, 0.1% Tween 20), loaded onto a 1 ml heparin column, and washed with wash buffer 3 (30 mM Hepes pH 7.4, 8% glycerol, 1 mM DTT, 180 mM NaCl). PrimPol was then eluted by step-gradient with elution buffer 2 (30 mM Hepes pH 7.4, 8% glycerol, 1 mM DTT, 600 mM NaCl) and diluted 2-fold with dilution buffer.

Purified proteins were aliquoted, frozen in liquid nitrogen and stored at –80ºC. Proteins were frozen only once before being used in the activity assay, unless indicated otherwise. Enzyme concentrations were adjusted as needed with elution buffer 1 or dilution buffer just before starting the reactions.

### Purification of PrimPol from *S*. *cerevisiae*

To create the yeast expression vector encoding for PrimPol with an N-terminal GST-tag fusion, the *PRIMPOL* gene was amplified by PCR and cloned in frame with GST using ClaI–SalI sites of the pRS424-GAL-GST-TRP plasmid [12]. The expression of *PRIMPOL* gene was under the control of a chimeric *GAL1-GAL10* promoter. PrimPol expression was carried out in the protease-deficient *S*. *cerevisiae* strain BJ2168 as described in [12] with modifications. Briefly, the yeast cells were transformed with pRS424-GAL-GST-TRP-PRIMPOL plasmid and plated on SCD selective glucose medium without tryptophan. Several colonies were streaked out onto a SCD plate and grown at 30ºC. After 2 days, the colonies were used to make 200 ml of overnight start culture in liquid SCR medium with raffinose (0.17% yeast nitrogen base, 0.5% ammonium sulphate, 20% filter sterilized raffinose, 0.1% drop-out mix (from a blended mixture of equal quantities of alanine, arginine, asparagine, aspartic acid, cysteine, glutamine, glutamic acid, glycine, histidine, isoleucine, lysine, methionine, phenylalanine, proline, serine, threonine, tyrosine, valine), 60 mg/l uracil, 60 mg/l adenine, 120 mg/l leucine, 50 mg/l kanamycin, pH 5.8). Next day, 4 liters of SCR medium were inoculated by start culture at 1:20 dilution and the cells were grown at 30ºC till OD_660_ >4. Then, 4 liters of rich YPRA medium (1% yeast extract, 2% g peptone, 1% raffinose, 60 mg/l adenine) were added and cells were grown for further 10 h. Induction of PrimPol expression was performed with 1% galactose, added as powder, and cells were grown for additional 6 h. Cells were harvested by centrifugation at 3500 g for 30 min and washed with ice cold water. Cells (typically 50–100 g from 8 liters of culture) were resuspended into equal amount of lysis buffer B (100 mM HEPES pH 7.8, 2% glycerol, 600 mM KCl, 40 mM K_2_HPO_4_/KH_2_PO_4_ pH 7.8, 1 mM DTT, 0.2% Tween 20, 20 μM pepstatin, 20 μM leupeptin, 5 mM benzamidine), frozen in liquid nitrogen by dropping and stored at– 80ºC. Yeast cells were disrupted with dry ice in laboratory blender with stainless steel chamber (Waring). When the powder was dissolved, PMSF was added to the suspension to a final concentration of 1 mM and glycerol was adjusted to a final concentration of 8%. GST-tagged PrimPol was purified from yeast cell lysates as described for *E*. *coli* and was concentrated to 1/10 volume with 30K Amicon Ultra Centrifugal filter unit.

### Purification of PrimPol from human cell culture

*PRIMPOL* gene was PCR-amplified and cloned into mammalian expression vector p3xFLAG CMV7.1 (Sigma-Aldrich) at the NotI and SalI sites to produce a recombinant protein with an N-terminal 3xFLAG tag. Recombinant PrimPol was produced in 200 ml suspension cultures of the FreeStyle™ 293-F cells in FreeStyle 293 Expression Medium (Gibco). Transient transfection of HEK 293-F cells in suspension cultures was performed using linear polyethylenimine (PEI) with MW 40,000 (PolySciences). For transfection, 200 μg of the expression plasmid and 0.6 ml of the PEI stock solution (1 mg/ml) were diluted in 6.6 ml Opti-Pro medium (Life Technologies). The mixture was incubated at room temperature for 25 min and then DNA/PEI solution was added to the cell culture flask dropwise. Cells were incubated with shaking at 125 rpm on a rotary shaker at 37°C in a humid atmosphere with 8% (v/v) CO_2_.

Cells were harvested at 72 h post-transfection and pelleted by centrifugation at 350 g for 5 min. After washing with ice-cold PBS, the cells were resuspended in 5 vol. of PBS with protease inhibitors (Roche) and sonicated. After sonication, the suspension was supplemented with 1% NP-40 and incubated on ice for 15 min and then centrifuged at 350 g for 5 min. The supernatant was additionally clarified by centrifugation at 17000 g for 10 min and agitated with 2 ml of anti-FLAG M2-agarose (Sigma-Aldrich) for 3 h at 4°C. The beads were packed into a disposable BioRad column, followed by washing with 20 ml buffer A (20 mM Tris-HCl, pH 7.4, 350 mM NaCl). PrimPol was eluted with 10 ml of 0.1 mg/ml 3xFLAG-peptide (Sigma-Aldrich) in buffer A. Purified protein was aliquoted and stored at– 80°C.

### DNA polymerase reactions

The DNA polymerase activity of PrimPol was analyzed in primer extension reactions with [^32^P]-5'-labeled, Cy5-5'-labeled or TET-5'-labeled oligonucleotide DNA substrates (S1 Table). The Cy5-labeled 12-mer primer was annealed to the 30-mer template and used to compare the activity of PrimPol protein purified from different *E*. *coli* strains, to estimate protein stability at different temperatures, pH and salt concentrations. [^32^P]-labeled or TET-labeled 25-mer primer was annealed to the 70-mer template and used for all other primer extension experiments.

Standard reactions were carried out in 10 or 20 μl total volume in the presence of 40 mM HEPES pH 7.0–7.25, 8% glycerol, 50 mM NaCl, 0.1 mg/ml bovine serum albumin, 10 mM MgCl_2_ and 200 μM dNTPs (GE Healthcare). The DNA templates were used at 100 nM concentration with the short 30-mer DNA substrate and at 25 nM with the long 70-mer DNA templates. PrimPol concentrations used were from 75 to 450 nM as specified on figure legends. In order to determine the optimal conditions for PrimPol DNA polymerase activity, HEPES was replaced with Tris-HCl, MES or Bis-Tris propane and рН varied from 5.0 to 8.0. The concentration of NaCl in reactions varied from 0 to 200 mM and the concentrations of MgCl_2_ and MnCl_2_ varied from 0.01 to 10 mM. The reactions were started by the addition of dNTPs or PrimPol and incubated at 37ºC for 10 min (or as specified). The reactions were terminated by the addition of an equal volume of loading buffer (95% formamide, 10 mM EDTA, 0.1% bromophenol blue). DNA products were resolved on 10–16% polyacrylamide gels containing 8 M urea and visualized using Typhoon 9400 («Amersham Bioscience») scanner. Gel quantification was done using ImageQuant image analysis software (GE Healthcare Life Sciences). The percentage of primer that was extended was calculated as the ratio between the signal of all extended products (i.e. all products longer than the primer) and the total lane signal. All experiments were repeated 2–4 times. The standard deviations were calculated and these values were used as error bars on all graphs presented in this study.

### Analysis of PrimPol stability under various temperature regimes, freeze-thaw cycles and mechanical stress

To test the stability of PrimPol in the DNA polymerase activity tests, reactions with 450 nM PrimPol from *E*. *coli* BL21(DE3) strain were pre-incubated together with DNA substrate at 0ºC, 25ºC, 30ºC, 35ºC, 37ºC or 40ºC for different periods of time (as indicated) and then used in primer extension assays. To check for proteolysis, PrimPol preparations were incubated at 37ºC for different periods of time and analyzed by SDS-PAGE on 10% gels using Coomassie staining.

In order to estimate the stability of PrimPol under freeze-thaw cycles, the PrimPol preparations from *E*. *coli* BL21(DE3) strain and from 293-F cells, diluted in elution buffer 1 (supplemented with 8% glycerol and HEPES) were frozen in liquid nitrogen, followed by melting on ice. This procedure was repeated 19 times. After each cycle, a 2 µl aliquot was taken and tested for DNA polymerase activity. To test the protein stability under mechanical stress, reaction mixtures containing PrimPol from *E*. *coli* BL21(DE3) strain were vortexed for 1–10 s before incubation at 37ºC.

## Results

### Effect of the reaction buffer composition on the DNA polymerase activity of PrimPol *in vitro*

**Fig 1:**
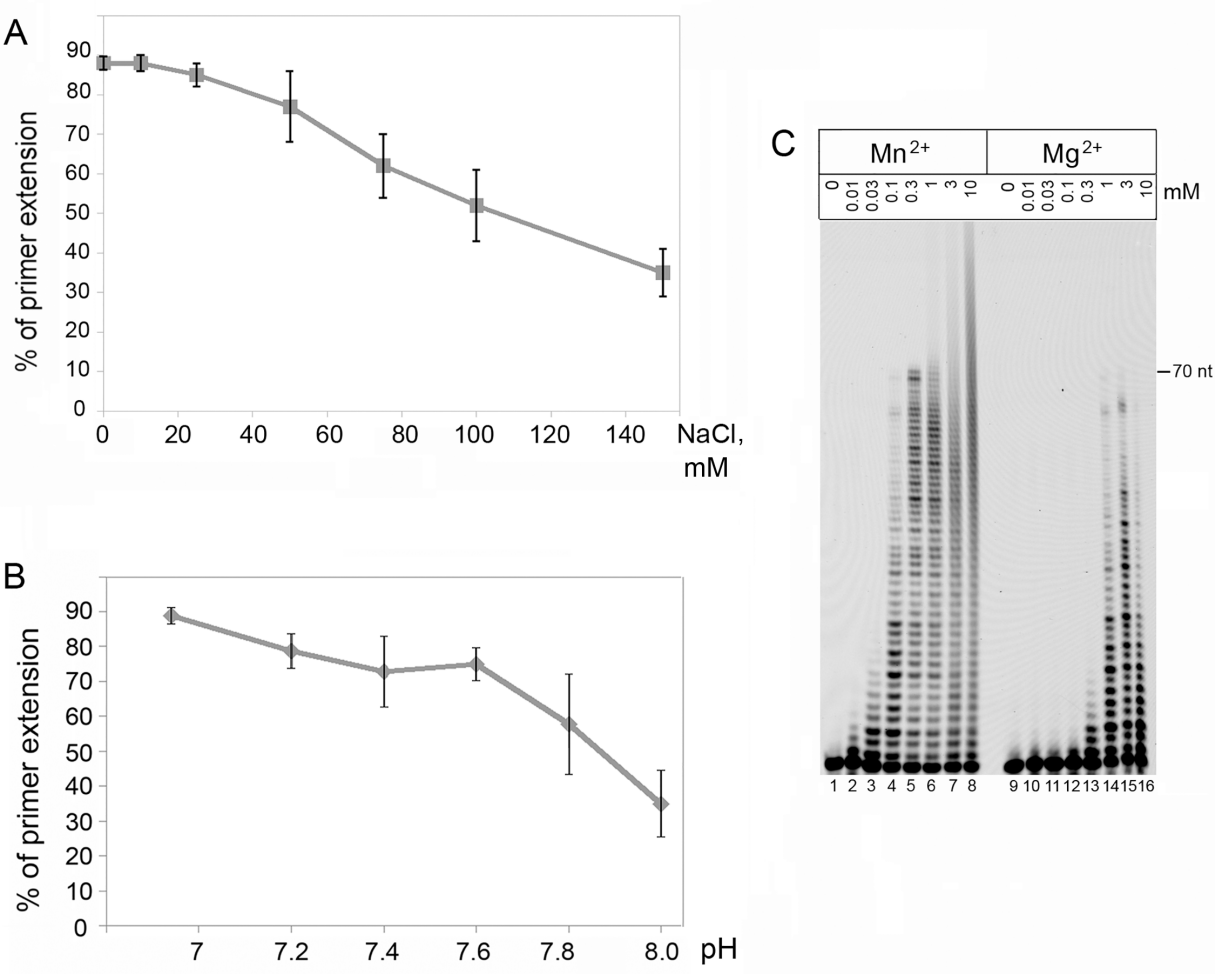
The reaction buffer composition affects the DNA polymerase activity of PrimPol *in vitro*. (A) the DNA polymerase activity of PrimPol at different NaCl concentrations. (B) the DNA polymerase activity of PrimPol at different pH. HEPES based buffer was used to test the DNA polymerase activity of PrimPol at different pH values. (C) the DNA polymerase activity of PrimPol at different MgCl_2_ and MnCl_2_ concentrations. 200 nM of PrimPol and 25 nM 70-mer DNA template were used in the reactions. All experiments were repeated three times.

The reaction buffer composition significantly affects the biochemical activity of enzymes. We tested the effect of different buffering agents, pH, Mg^2+^/Mn^2+^ concentrations and ionic strength on the DNA polymerase activity of PrimPol (Fig 1). The highest PrimPol activity was observed when reaction buffer was not supplemented with salt (Fig 1A). Importantly, PrimPol demonstrated the highest DNA polymerase activity at relatively low pH (6.0–7.0) in both HEPES-based buffer (Fig 1B) as well as Bis-Tris-propane and Tris-based buffers (Figs 1B and S1). In MES-based buffer, which has a maximum buffering capacity at the low pH range (5.5–6.7), pH 6.0 was found to be optimal for PrimPol primer extension activity (S1A and S1B Fig). However, at pH 7.0, which is more physiologically relevant, the DNA polymerase activity of PrimPol was modestly higher in HEPES- and Tris-based buffers (S1A and S1C Fig). The addition of BSA to the reaction mixture did not significantly influence the DNA polymerase activity of PrimPol (S1C Fig).

Previous studies revealed that PrimPol can utilize both Mn^2+^ and Mg^2+^ ions as cofactors for DNA polymerization [1, 2, 6, 8]. As shown in Fig 1C, however, Mn^2+^ stimulates PrimPol’s polymerase activity at at least 10-fold lower concentrations than Mg^2+^, which is in agreement with a previous report [2]. At higher Mn^2+^ concentrations (3–10 mM, lanes 7–8), we also observed primer extension products longer than 70 nt, indicating either terminal transferase or template re-aligning capabilities of PrimPol. No such activity was observed at any of the Mg^2+^ concentrations tested. Instead, a weak signal, corresponding to the expected full-length product, is visible at 3 and 10 mM MgCl_2_ concentrations used. These data are in agreement with previously reported results [13,14].

### PrimPol is stable under multiple freeze-thaw cycles, but quickly loses activity at the physiological temperature

**Fig 2:**
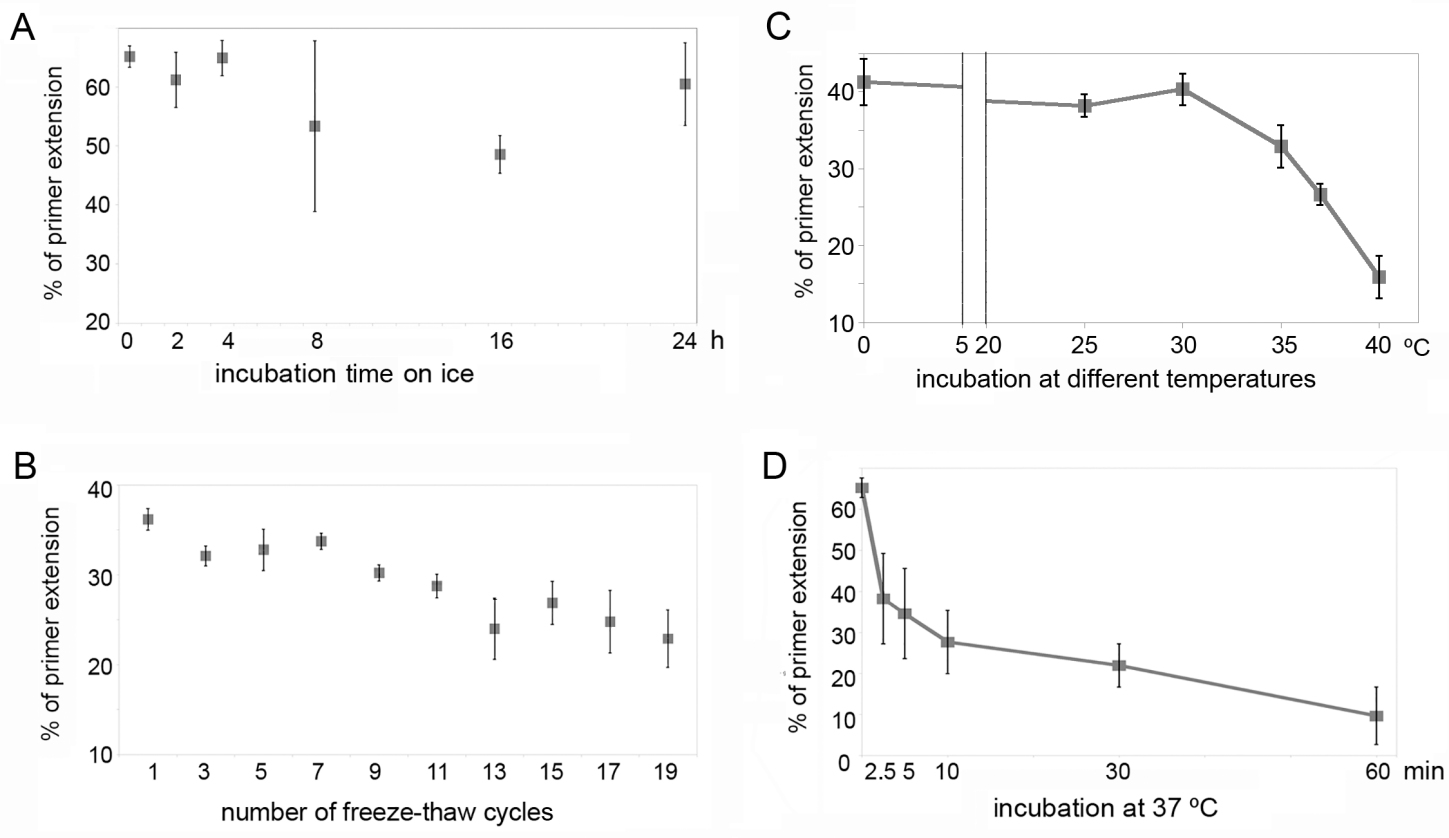
The stability of the PrimPol’s catalytic activity *in vitro*. (A) the DNA polymerase activity of PrimPol after the indicated time pre-incubation on ice. (B) the DNA polymerase activity of PrimPol after the indicated amount of freeze-thaw cycles. (C) the DNA polymerase activity of PrimPol after 30 min incubation at 0ºC, 25ºC, 30ºC, 35ºC, 37ºC and 40ºC. (D) the DNA polymerase activity of PrimPol after incubation at 37ºC. 450 nM of PrimPol and 100 nM 30-mer DNA template were used in the reactions. Experiments were repeated two times in A and B and three times in C and D.

We analyzed the stability of the PrimPol’s catalytic activity under various conditions: exposure to multiple freezing-refreezing events and incubation at different temperatures. PrimPol retains DNA polymerase activity after prolonged incubation at low temperatures, as the activity was reduced by only 7% after 24-h exposure at 0ºC (Fig 2A). Similarly, under freeze-thaw cycles PrimPol remains largely active with the DNA polymerase activity reduced by 21% after 10 freezing-refreezing cycles and by 41% after 19 freeze-thaw cycles (Fig 2B). Therefore, on average, each cycle reduces the polymerase activity for only 2%.

PrimPol was also stable under 30 min incubations at 25°C and 30°C (Fig 2C). In contrast, we found that the catalytic activity of PrimPol was significantly reduced after 30 min incubation at 35°C– 40°C, despite the presence of protective BSA protein in the reaction mixture (Fig 2D). The estimated half-life time of PrimPol activity at the physiological temperature 37ºC is only ~ 13 min at the assay conditions. The loss of DNA polymerase activity was not associated with visible proteolysis (data not shown). Additionally, we found that PrimPol is not stable under mechanical stress as 3–10 sec of vortexing lead to a marked loss of the DNA polymerase activity (S2 Fig).

### Common *E*. *coli* expression strains provide high yield of PrimPol proteins with similar catalytic activity

*E*. *coli* is the most widely used host for production of recombinant proteins. Strains BL21(DE3), BL21(DE3)-pRIL and Rosetta(DE3)-pRARE were used in previous studies for PrimPol production at 15ºC– 30ºC [1, 2, 6, 8] (S2 Table). In an attempt to increase the yield and/or activity of purified protein, we also included in this study another DE3 derivative strain, ArcticExpress(DE3)-pRIL, in addition to BL21(DE3) and Rosetta 2(DE3)-pRARE2. To improve protein expression at low temperatures (12ºC in this study), ArcticExpress(DE3)-pRIL strain of *E*. *coli* also expresses chaperons from the psychrophilic bacterium *Oleispira antarctica*.

**Fig 3:**
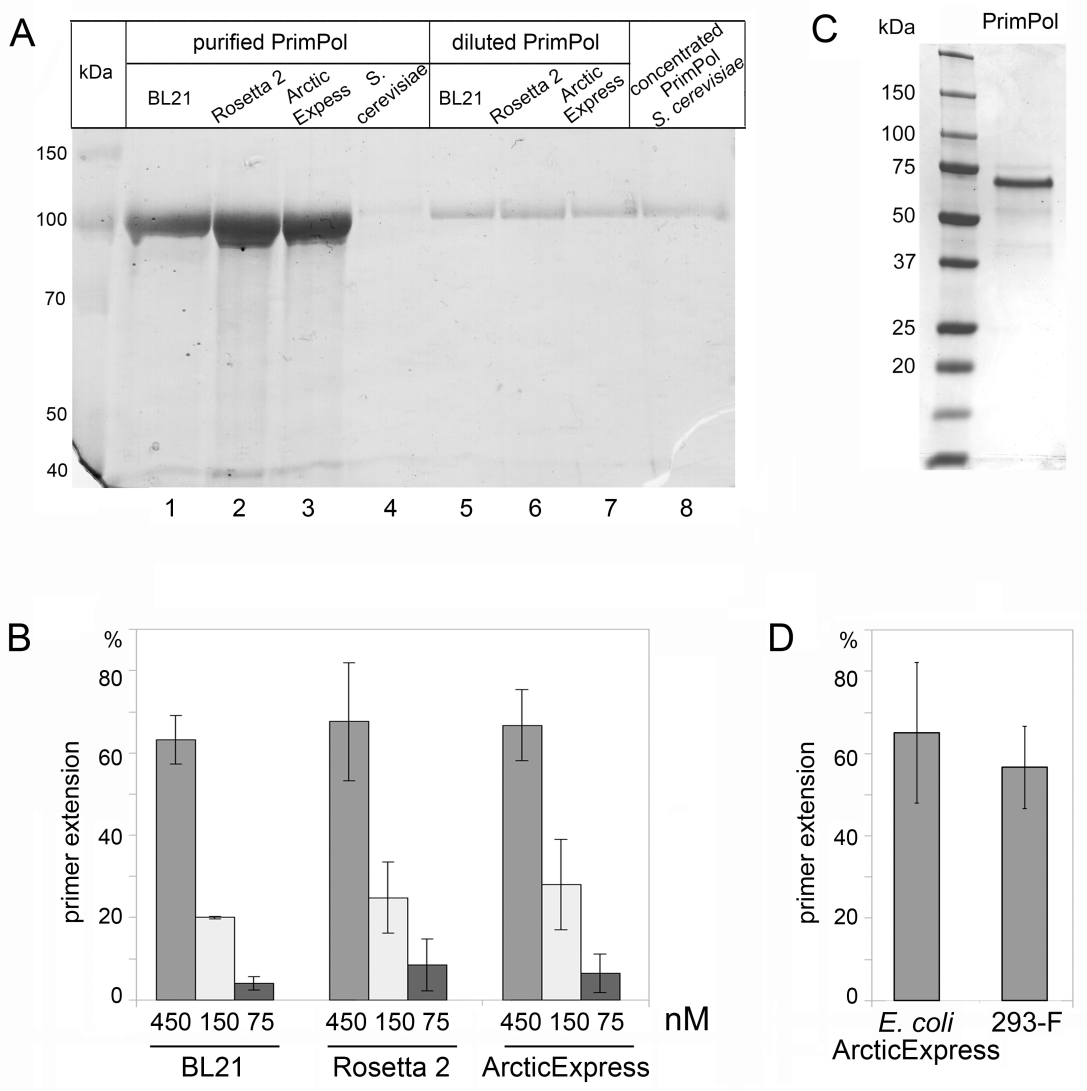
Purification and the catalytic activity of PrimPol from different expression systems. **(**A) purified preparation of GST-tagged PrimPol from different *E*. *coli* strains and *S*. *cerevisiae*. Coomassie staining. (B) the comparison of the DNA polymerase activity of PrimPol purified from different strains of *E*. *coli*. 75–450 nM of GST-tagged PrimPol and 100 nM 30-mer DNA template were used in the reactions. (C) purified preparation of PrimPol from human FreeStyle 293-F cells. Coomassie staining. (D) the comparison of the DNA polymerase activity of PrimPol purified from ArcticExpress(DE3)-pRIL *E*. *coli* cells and human FreeStyle 293-F cells. 200 nM of PrimPol and 25 nM 70-mer DNA template were used in the reactions. Experiments (B and D) were repeated four times.

To facilitate purification, PrimPol proteins were fused at the N-terminus with a GST-tag. GST-tag promotes protein solubility and protects proteins from proteolytic degradation in *E*. *coli* [15]. Proteins were purified using one step affinity chromatography and were judged > 95% pure by SDS-PAGE (Fig 3A). Despite the differences among the *E*. *coli* strains used, similar amounts of soluble human PrimPol protein were obtained, with yields exceeding 1 mg per liter of LB medium with all tested strains (Table 2). We have also compared the DNA polymerase activity of PrimPol proteins purified from different strains of *E*. *coli* in a primer extension assay with increasing enzyme concentrations (Fig 3B). No significant differences were observed in the activity of PrimPol preparations, confirming no benefits of expression at low temperature and co-expression with cold-adapted chaperones in the ArcticExpress(DE3)-pRIL strain.

**Table 2:** 

Host organism	Strain	Temperature of cell growth during expression	Protein yield [µg/l of cell culture]
*E*. *coli*	BL21(DE3)	19ºC	1050
	Rosetta 2 (DE3)-pRARE	19ºC	1350
	ArcticExpress(DE3)-pRIL	12ºC	1120
*S*. *cerevisiae*	BJ2168	30ºC	~ 1
human cell line	FreeStyle 293-F	37ºC	1600

### *S*. *cerevisiae* cells provide low yield of recombinant human PrimPol

Budding yeast *S*. *cerevisiae* can be easily cultured and may provide some benefits for expression of human proteins since yeast cells have protein-folding and post-translational modification mechanisms related to those found in mammalian cells [16]. We produced and purified full-length PrimPol from *S*. *cerevisiae* using a protocol similar with protocols described for human Pol ι and yeast Pol ζ and Rev1 [17–19]. Unexpectedly, the yield of full-length human PrimPol from *S*. *cerevisiae* was very low (Table 2). The average yield of protein was ~ 1 μg from 1 liter of yeast cell culture in three independent purifications. After concentrating on an Amicon Filter Unit, the concentration of the protein purified from yeast cells was still only 225 nM (Fig 3A, lane 8), which was too low to reliably measure the catalytic activity of human PrimPol produced in yeast.

### PrimPol production in human cell line provides high yield

Transient expression in human cell culture is a powerful alternative for production of human enzymes including large and unstable multisubunit human DNA polymerases complexes [20]. We overexpressed PrimPol fused with the N-terminal 3xFLAG-tag in suspension culture of FreeStyle 293-F cells. The highest protein expression was observed 72 hours after transfection (data not shown). The yield of PrimPol from this expression system was the highest among the tested expression systems (Table 2) (Fig 3C). Importantly, PrimPol purified from human cells demonstrated similar catalytic activity and stability as was measured for PrimPol purified from *E*. *coli* (Figs 3D and S3).

## Discussion

### The overall low catalytic activity of human PrimPol

Human cells contain a variety of DNA polymerases that differ in their functions and fidelity of DNA synthesis. PrimPol is a recently discovered human DNA polymerase, which also possesses the primase activity [1–3, 7, 9]. However, many aspects of PrimPol’s biochemical activities and its function in the cells remain unknown.

Several groups carried out biochemical studies of human PrimPol produced in *E*. *coli* [1–3, 6, 8, 21]. It was shown that human PrimPol preparations possess relatively poor biochemical activity *in vitro*, and have low affinity for DNA and dNTPs. In particular, in the presence of Mg^2+^ ions, the observed dissociation constant for DNA (*K*_d_)_(DNA)_ was 240–8000 nM and *K*_M_ for correct dNTPs incorporation was 840 μM [6, 8, 11]. In contrast, *K*_M_ for correct dNTPs incorporation did not exceed 1.1 μM for mitochondrial Pol γ and 2.6–260 μM for other TLS human DNA polymerases (Pol β, Pol λ, Pol η, Pol ι and Pol κ) [22]. Excess of PrimPol over DNA template and high concentrations of dNTPs were therefore used in most biochemical studies of PrimPol to follow its activity (S2 Table) [6].

The composition of the reaction buffer, impaired protein folding in host microorganism or protein instability may have contributed to the poor catalytic activities observed. We tested how expression in different systems and reaction conditions affected the PrimPol activity and determined the enzyme stability under different conditions. The data reported here will assist to standardize protocols for future PrimPol studies.

### The effect of the reaction buffer composition on the DNA polymerase activity of PrimPol

The composition of the reaction buffer used (*e*.*g*. pH, metal-cofactor and salt concentrations) differed considerably in previous studies (S2 Table). We tested different reaction buffer compositions and showed that low salt conditions and pH 6.0–7.0 in combination with 3 mM Mg^2+^ or 0.3–10 mM Mn^2+^ ions support the highest DNA polymerase activity of human PrimPol *in vitro*.

The observed inhibition of the DNA polymerase activity of PrimPol by salt is similar to other DNA polymerases [23, 24]. Interestingly, human PrimPol demonstrates lower-than-physiological pH value preference. In MES-based buffer, the highest DNA polymerase activity of PrimPol was observed at 5.75–6.25. This is different from many other enzymes, including DNA polymerases, which have the pH optimum near the physiological pH of 7.5 or even higher. For example, *E*. *coli* Pol I as well as *S*. *cerevisiae* and human Pol η, Pol λ and Pol α all have the pH optimum at 8–9 in Tris-HCl buffer [23, 25–27]. Interestingly, PrimPol has the predicted pI at 5.07–5.18. It is possible that the negative charge of some functional protein residues at high pH may interfere with its catalytic activity (*e*.*g*. DNA binding). The choice of the buffering agent also affects the activity of PrimPol. At pH 7.0, HEPES-based buffer supports the highest DNA polymerase activity of PrimPol.

Interestingly, the pH and salt preference of GST-tagged PrimPol differs from those of the untagged protein. 50–100 mM NaCl and pH 7.2–7.4 were found to be ideal for the DNA polymerase activity of GST-PrimPol (S4 Fig). The higher salt concentrations may be required to prevent GST-tagged PrimPol aggregation *in vitro*. It is likely that GST-tag could also influence PrimPol’s pI or charge distribution.

Post-translational modifications or interaction with accessory factors might also influence the pI or charge distribution of PrimPol and this could potentially play a role in the regulation of its activity *in vivo*.

### Stability of PrimPol *in vitro*

As shown in Fig 2, PrimPol can be stored on ice for 16–24 h and frozen-refrozen several times in HEPES-based buffer supplemented with 8% glycerol without considerably affecting its DNA polymerase activity. However, the enzyme is very sensitive to heating and mechanical shearing (at the assay conditions). A significant reduction of PrimPol activity was observed after protein incubation at ≥ 35 ºC. The DNA polymerase activity dropped by 50% after 13 min of incubation at 37ºC, however the observed loss of activity was not accompanied by proteolysis. Thus, the heat inactivation can be a result of small irreversible changes in the protein conformation due to local thermal denaturation or aggregation. Exposure of PrimPol to elevated temperatures and vortexing should therefore be avoided, both during the purification and manipulation with purified protein. Ideally, the reaction time at 37ºC during DNA biochemical tests should not exceed 5–10 min. Alternatively, 30ºC might be used to prevent the rapid loss of the catalytic activity of PrimPol *in vitro*.

### The optimal expression host for production of recombinant human PrimPol

In this work, we tested three *E*. *coli* strains, a *S*. *cerevisiae* strain and a human suspension cell culture for PrimPol production.

Due to simplicity of expression and purification procedures, high protein production levels and low costs of cell cultivation, protein expression in *E*. *coli* is the most common way for recombinant protein production. However, the lack of eukaryotic post-translation machinery and rapid protein accumulation can sometimes promote misfolding of eukaryotic proteins, resulting in loss of their biochemical activity [28, 29]. A common alleviation of rapid protein accumulation can be achieved by lowering the temperature of cultivation, which slows down the rate of folding, thereby decreasing the hydrophobic interactions involved in protein self-aggregation and often improving the solubilization of the protein [30]. Another strategy to prevent protein aggregation and to increase the catalytic activity of eukaryotic enzymes is the co-expression of target protein with chaperons [29, 31]. With these considerations in mind, we carried out the production of human PrimPol in three different *E*. *coli* strains. BL21 is the most common host strain as it enables high-level recombinant protein expression. Rosetta 2-pRARE is BL21-derivative strain expressing tRNAs for seven codons rarely used in bacteria and designed to enhance the expression of eukaryotic proteins. ArcticExpress(DE3)-pRIL strain contains extra copies of tRNA genes for rare *E*. *coli* codons and also encodes the cold-adapted chaperons Cpn10 and Cpn60, which aid in protein folding at low temperatures. In our experiments, all strains provided good yield of human PrimPol with similar DNA polymerase activities, indicating that additional tRNAs for rarely used codons, low temperature of expression and co-expression with chaperones are not necessary for production of active PrimPol protein.

Eukaryotic expression systems offer some advantages for human recombinant proteins production. In this work, we for the first time report the production of full-length human PrimPol from yeast and human cells. For instance, many human DNA polymerases (*e*.*g*. Pol κ, Pol η, Pol ι), which were difficult to express and purify as full-length active proteins in *E*. *coli* along with unstable DNA polymerases variants, were successfully produced and isolated from yeast [17, 32–34]. Recently, *S*. *cerevisiae* cells were used for production and purification of the N-terminal domain of human PrimPol for crystallographic studies [35]. Despite this, the yield of full-length recombinant PrimPol protein from *S*. *cerevisiae* in our experiments was markedly lower when compared to *E*. *coli* cells. Codon-optimization of human *PRIMPOL* gene might be necessary to increase the protein yield from *S*. *cerevisiae* cells.

Human cells provide the natural environment for correct protein folding and post-translational modifications which ensure the highest catalytic activity of human proteins. Human FreeStyle 293-F cells were found to be a good expression system for the production of recombinant human PrimPol and provided high yield of active protein. However, the production of PrimPol in human cells did not improve the catalytic activity compared to expression in *E*. *coli* or its stability. These data indirectly suggest that PrimPol in *E*. *coli* cells undergoes correct folding.

The purification protocol of PrimPol from human cell lines presented in this study, however, opens up new possibilities for *in vitro* study of this protein. While we didn’t observe significant differences in the polymerase activities between PrimPol produced in human and bacterial cells, the post-translational modifications of PrimPol produced in human cell line could be of importance for other functional PrimPol studies, *e*.*g*. protein-protein interactions. The purification protocol of PrimPol from human cell lines presented in this study could be used for fractionation of nuclear and mitochondrial subpopulations of PrimPol. In turn, this would make it possible to *in vitro* study compartment-specific PrimPol variants.

## Conclusions

In this work we compared the effect of different reaction buffer compositions on PrimPol DNA polymerase activity, estimated its enzymatic stability at different conditions and tested different expression systems for its production. The presented data will enable researchers to improve experimental design and will assist to understand results obtained from future experiments. Our data also show that both human and *E*. *coli*, but not *S*. *cerevisiae*, expression systems provide high protein yields and robust biochemical activity of recombinant full-length human PrimPol. Taking into consideration the simplicity and the lower costs of bacterial cultivation comparison with human cells, we suggest that common *E*. *coli* expression strains are the best choice for recombinant human PrimPol production for biochemical studies of its activity. On the other hand, PrimPol from human cell lines might be preferred for more complex studies including analysis of its interactions with various partner proteins.

## Supporting information

S1 Table Oligonucleotide sequences used in this study. (DOC)

S2 Table Expression conditions and buffer compositions used in PrimPol studies. (DOC)

S1 Fig The DNA polymerase activity of PrimPol with different buffers and pH. 200 nM of PrimPol and 25 nM 70-mer DNA template were used in the reactions. Experiments were repeated two times.
(JPG)

S2 Fig The stability of PrimPol under mechanical stress. Reaction mixtures containing PrimPol were vortexed for 1–10 seconds before incubation at 37ºC. 200 nM of PrimPol and 25 nM 70-mer DNA template were used in the reactions. Experiment was repeated three times.
(JPG)

S3 Fig Purification and stability of the catalytic activity of PrimPol from 293-F cells. (A) silver-stained gel analysis of PrimPol from *E*. *coli* cells (without GST-tag) and of 3xFLAG-tagged PrimPol from 293-F cells. (B) DNA polymerase activity of PrimPol purified from 293-F cells after incubation on ice. (C) the DNA polymerase activity of PrimPol purified from 293-F cells after the indicated amount of freeze-thaw cycles. 450 nM of PrimPol and 100 nM 30-mer DNA template were used in the reactions. Experiments were repeated three times in B.
(JPG)

S4 Fig **The DNA polymerase activity of GST-tagged PrimPol at different NaCl concentrations (A) and pH (B).** HEPES based buffer was used in reactions. Experiments were repeated three times in A and two times in B.
(JPG)
