# Supplementary material for: Optimization of the expression, purification and polymerase activity reaction conditions of recombinant human PrimPol
Source: PLoS One. 2017 Sep 13;12(9):e0184489. doi: 10.1371/journal.pone.0184489 (PMC5597260; doi:10.1371/journal.pone.0184489)
Supplement: S1 Table — (DOC) [file pone.0184489.s001.doc]

**S1 Table. Oligonucleotide sequences used in this study**

| Oligonucleotide name | Sequence (5' 🡪 3') |
| --- | --- |
| Cy5-labeled 12-mer primer | CAGAGATACTAC |
| 30-mer template | GAGCAGTCGCACAGGTAGTATCTCTGTGAC |
| TET-labeled or [^32^P]-labeled 25-mer | ATAGGGGTATGCCTACTTCCAACTC |
| 70-mer template | GAGGGGTATGTGATGGGAGGGCTAGGATATGAGGTGAGTTGAGTGGAGTTGGAAGTAGGCATACCCCTAT |
