# Supplementary material for: Optimization of the expression, purification and polymerase activity reaction conditions of recombinant human PrimPol
Source: PLoS One. 2017 Sep 13;12(9):e0184489. doi: 10.1371/journal.pone.0184489 (PMC5597260; doi:10.1371/journal.pone.0184489)
Supplement: S2 Table — (DOC) [file pone.0184489.s002.doc]

**S2 Table. Expression conditions and buffer compositions used in PrimPol studies**

| *E. coli* strain | Protein expression conditions | tag | pH of reaction buffer | Metal ions | salt | reaction time | DNA/PrimPol concentrations | article |
| --- | --- | --- | --- | --- | --- | --- | --- | --- |
| not indicated | 0.2 mM IPTG at 17ºC for 16 h | N-terminal GST | pH 7.5 | 8 mM MgCl_2_ | 120 mM NaCl | 30 min at 37ºC | not indicated | [3] |
| BL21-pRIL(DE3) | 1 mM IPTG at 30ºC for 2.5 h | N-terminal GST and 6xHIS | pH 7.5 | 1 mM Mn^2+^ * | 75 mM NaCl | 60 min at 30ºC | 2.5 nM DNA and 200 nM PrimPol or 1.5 nM and 400 nM PrimPol | [2] |
| BL21(DE3) | 0.4 mM IPTG at 16ºC, overnight | N-terminal 6xHIS | pH 7.0 | 10 mM MgCl_2_ | 10 mM NaCl | 2-30 min at 37ºC | 20 nM DNA and 50 nM PrimPol | [1] |
| SHuffle Express cells | 0.4 mM IPTG at 16ºC, overnight | N-terminal 6xHIS | pH 7.9 | 10 mM MgAc | 50 mM KAc | 1-10 min at 37ºC | 20 nM DNA and 100 nM PrimPol | [21] |
| Rosetta(DE3) | auto-induction based on diauxic growth, 15ºC for 40 h | SUMO | pH 7.5 | 10 mM  MgCl_2_** | 100 mM KCl | 37ºC | 2500 nM DNA and 250 nM PrimPol | [6] |
| BL21-pRIL(DE3) | 1 mM IPTG at 30ºC for 2.5 h | N-terminal 6xHIS | pH 7.0 | 10 mM MnCl_2_ | not indicated | rapid quench kinetics, 37ºC | 300 nM DNA and 10 000 nM PrimPol | [8] |

* various concentrations of Me^2+^ ions were tested. PrimPol demonstrated the highest activity at 1 mM Mn^2+^. PrimPol activity at 10 mM Mg^2+^ was less efficient than at 0.01-1 mM Mg^2+^.

** various concentrations of Me^2+^ ions were tested. PrimPol demonstrated the highest activity at 10 mM Mn^2+^. PrimPol activity at 10 mM Mg^2+^ was more efficient than at 0.1 mM Mg^2+^.
