# Supplementary figures and images for: Optimization of the expression, purification and polymerase activity reaction conditions of recombinant human PrimPol
Source: PLoS One. 2017 Sep 13;12(9):e0184489. doi: 10.1371/journal.pone.0184489 (PMC5597260; doi:10.1371/journal.pone.0184489)

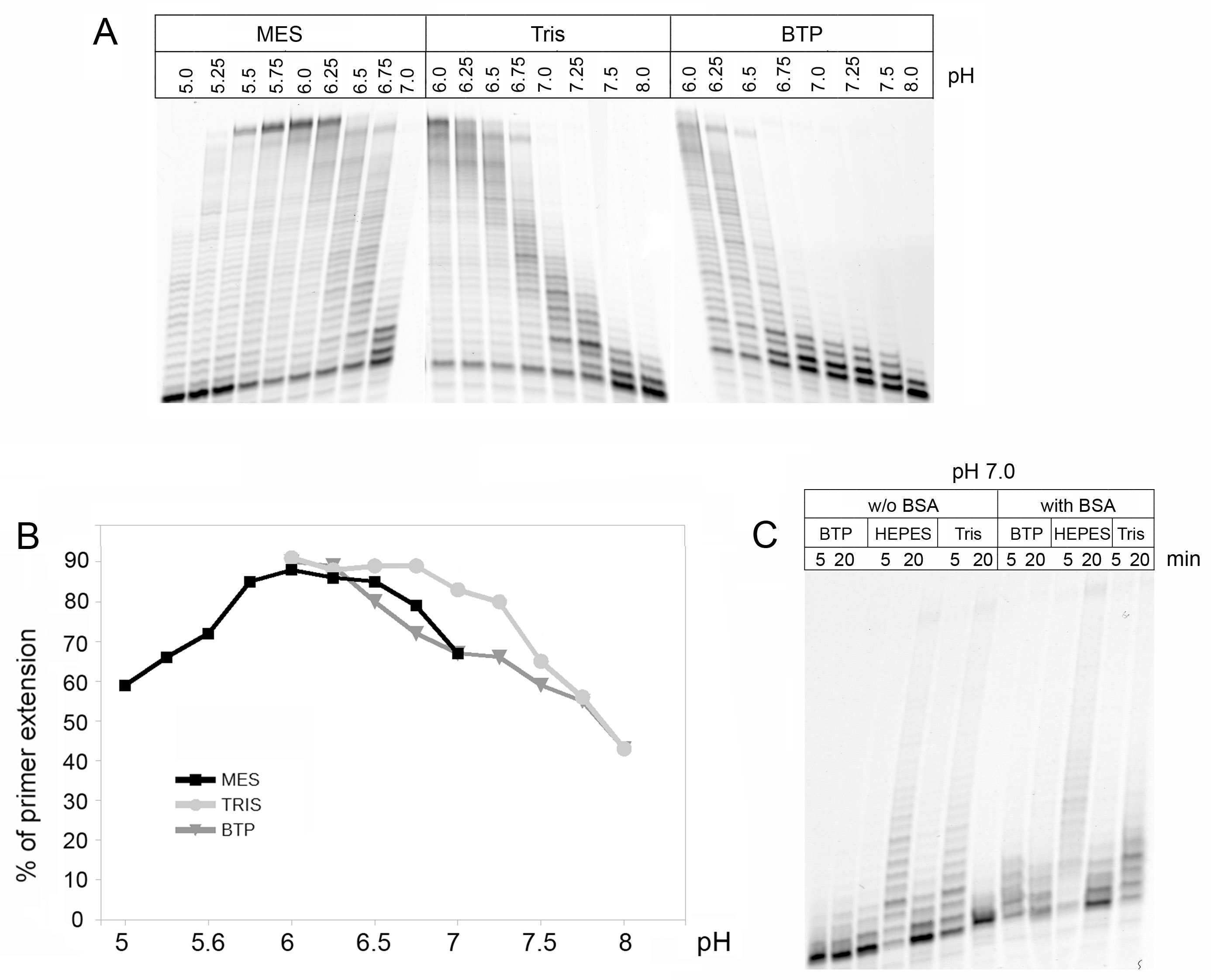

Supplement: S1 Fig — 200 nM of PrimPol and 25 nM 70-mer DNA template were used in the reactions. Experiments were repeated two times. (JPG) [file pone.0184489.s003.jpg]

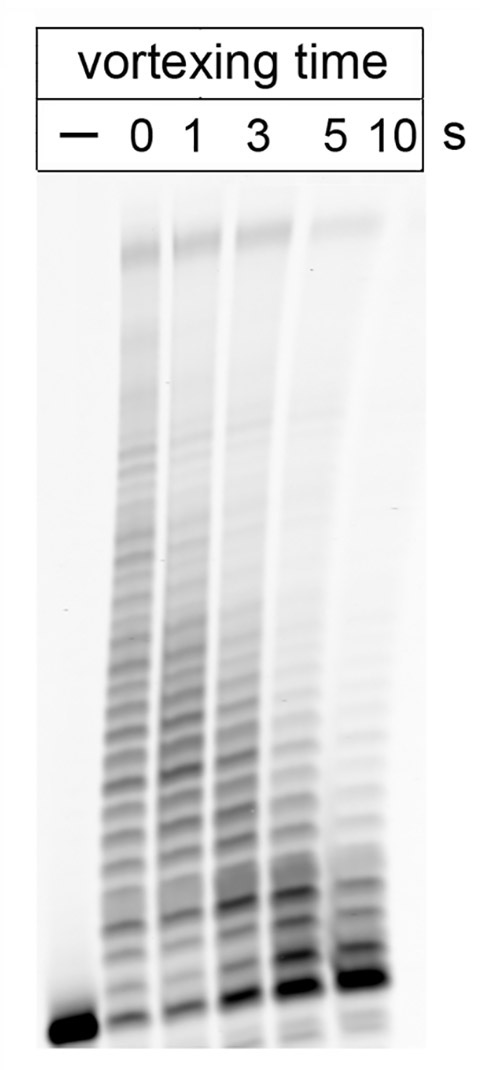

Supplement: S2 Fig — Reaction mixtures containing PrimPol were vortexed for 1–10 seconds before incubation at 37ºC. 200 nM of PrimPol and 25 nM 70-mer DNA template were used in the reactions. Experiment was repeated three times. (JPG) [file pone.0184489.s004.jpg]

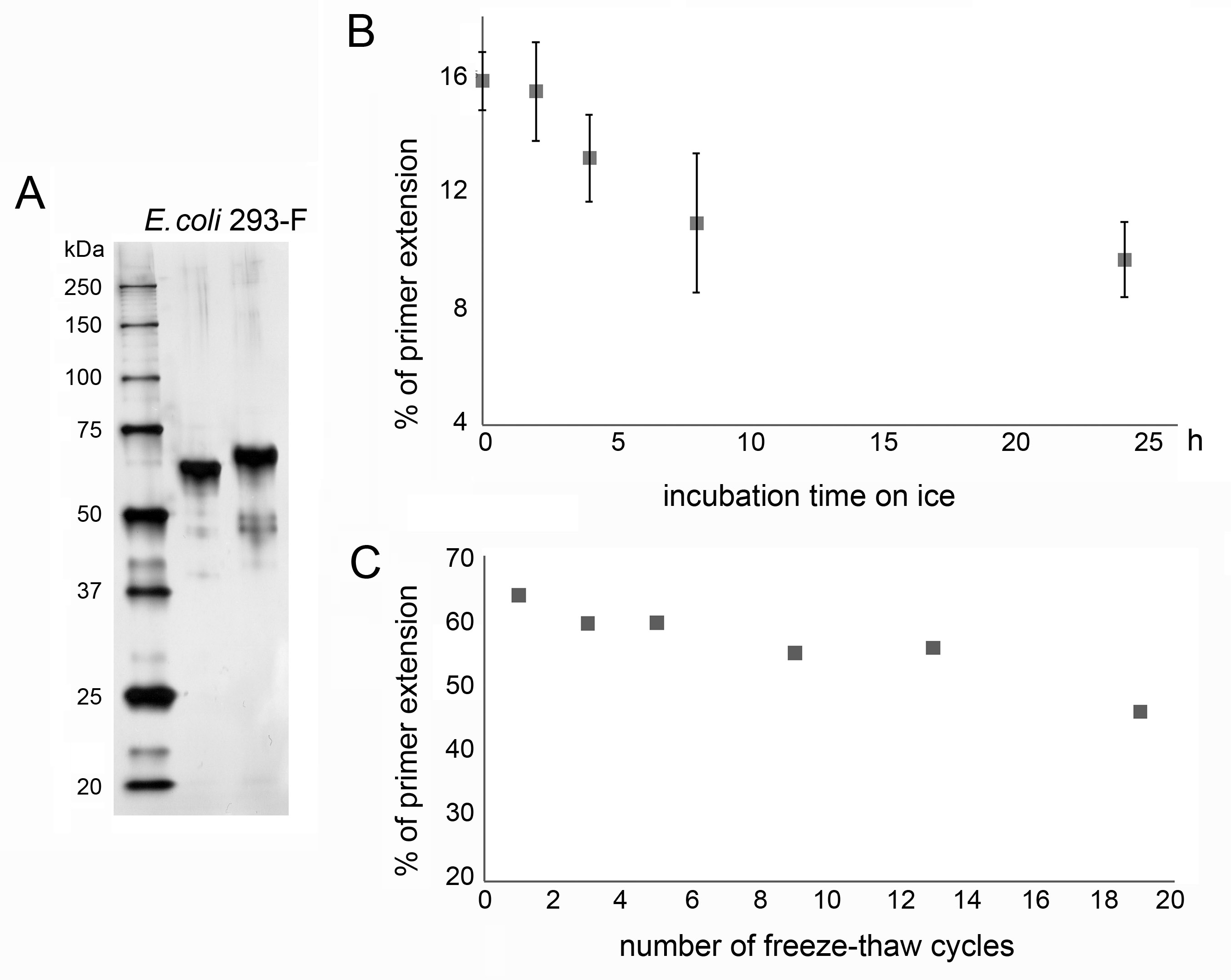

Supplement: S3 Fig — (A) silver-stained gel analysis of PrimPol from E. coli cells (without GST-tag) and of 3xFLAG-tagged PrimPol from 293-F cells. (B) DNA polymerase activity of PrimPol purified from 293-F cells after incubation on ice. (C) the DNA polymerase activity of PrimPol purified from 293-F cells after the indicated amount of freeze-thaw cycles. 450 nM of PrimPol and 100 nM 30-mer DNA template were used in the reactions. Experiments were repeated three times in B. (JPG) [file pone.0184489.s005.jpg]

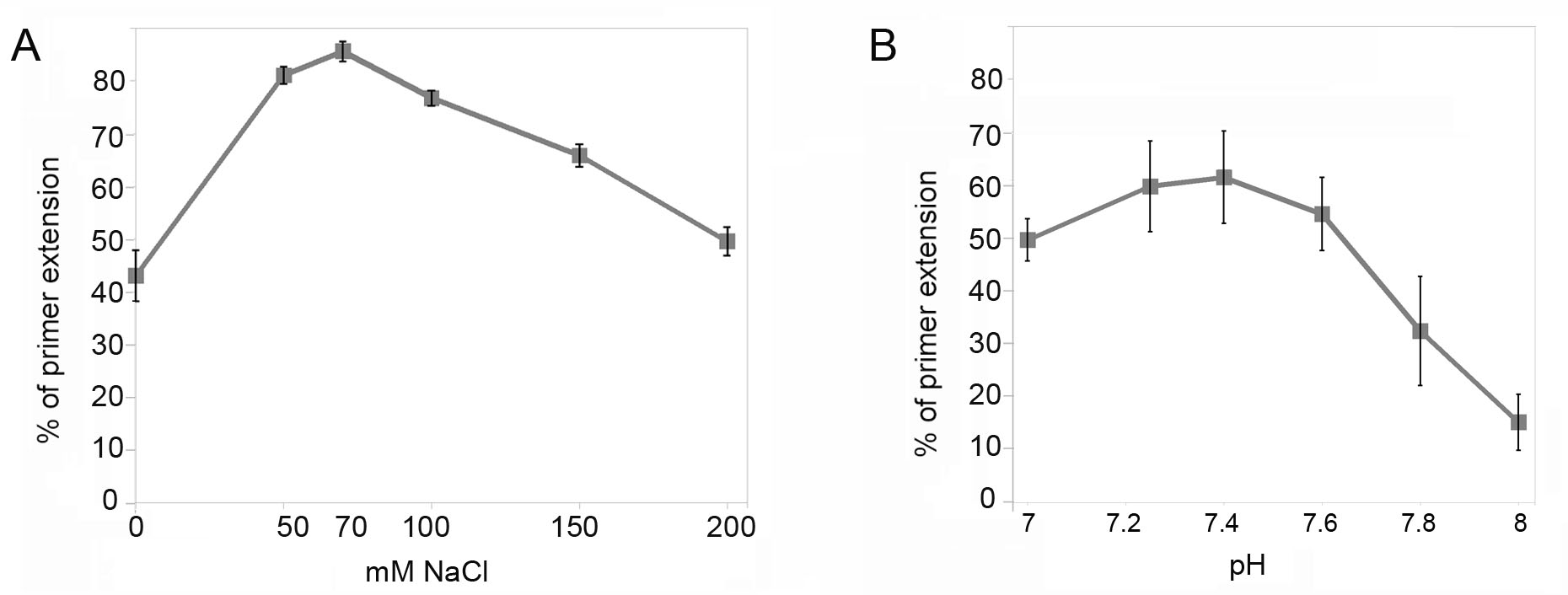

Supplement: S4 Fig — The DNA polymerase activity of GST-tagged PrimPol at different NaCl concentrations (A) and pH (B). HEPES based buffer was used in reactions. Experiments were repeated three times in A and two times in B. (JPG) [file pone.0184489.s006.jpg]
